# Instability Pattern Formation in a Liquid Metal under High Magnetic Fields

**DOI:** 10.1038/s41598-017-02610-6

**Published:** 2017-05-22

**Authors:** Jun Wang, Jinshan Li, Hongchao Kou, Eric Beaugnon

**Affiliations:** 1 State Key Laboratory of Solidification Processing, Northwestern Polytechnical University, Xi’an Shaanxi, 710072 China; 2 Univ. Grenoble Alps, LNCMI, F-38000 Grenoble, France; 3 CNRS, LNCMI, F-38000 Grenoble, France

## Abstract

Magnetic field can generate interface instability when some liquids are put close to magnetic field. A well-known interface instability is called Rosensweig instability or normal field instability. Here we report that pure liquid Co can be highly undercooled close to its Curie temperature in strong magnetic field with very high magnetization and exhibiting unique morphology instability called the normal field instability. To obtain such unique instability pattern, the sample size, undercooling and magnetic field intensity need fulfill certain condition. In the present study, we have studied the required condition for obtaining normal field instability. The magnetization of the undercooled liquid Co is measured in a wide temperature range with different magnetic field intensities and calculated as a function of undercooling and field intensity. The critical size and critical magnetization for the normal field instability are calculated with the changing temperature and field intensity. Then the required conditions including the critical size, the minimum undercooling and field intensity for the existence of the instability pattern formation are determined.

## Introduction

Magnetic fields can induce instability pattern in many liquid systems^1, 2^. A special one called Rosensweig instability or normal field instability is widely observed for very many magnetic fluids^2, 3^, which is formed in ferrofluids when subjected to a vertical oriented and uniform magnetic field due to interacting combinations of dipolar, interfacial and geometric influences^4, 5^.

Ferromagnetism has been known exist only in solid state since no substance is known whose Curie temperature (*T*_C_) exceeds its melting point (*T*_m_)^6^. Thus, the Rosensweig instability is thought only exist in ferrofluids which are colloidal liquids made of nanoscale ferromagnetic, or ferrimagnetic particles suspended in a carrier fluid (usually an organic solvent or water)^4^. Then the size and volume fraction of the particles have very strong effect on the magnetic properties of the liquid and finally change the pattern when subjected to a magnetic field^7^.

Theoretical investigations based on the Heisenberg model show that it is possible for long range magnetic ordering to exist in homogenous liquid^8, 9^. However, except for the ferrofluids, the existence of Rosensweig instability in pure liquid system is only observed in liquid oxygen^10^. A new idea considering undercooling technique was adopted for some special alloy system (such as Co-Pd^11^ and pure Co^12, 13^) and ferromagnetism or strongly magnetized liquid was achieved by directly undercool the alloy below or approach the transition temperature of the magnetic state (*T*_C_(L))^6, 8, 12–15^. The transition from a paramagnetic to a ferromagnetic state of long range magnetic order was found exist when the temperature of the undercooled alloy approaches *T*_C_(L) and the undercooled liquid follows the Curie-Weiss law^8^. The Rosensweig instability pattern in pure metallic liquid was first evidenced in supercooled liquid Co due to the strong magnetization caused by high magnetic field^13^. Thus question arises under what conditions the Rosensweig instability can be observed in pure liquid metallic melt. To solve this problem, in the present paper, the magnetization of liquid Co was calculated based on directly measurement through a wide temperature range from large overheating to deep supercooling in various magnetic field intensities. The conditions for the existence of Rosensweig instability are determined and discussed by comparison with the critical magnetization and size for normal field instability.

## Results

### Supercooling of Co in high magnetic field

**Figure 1:**
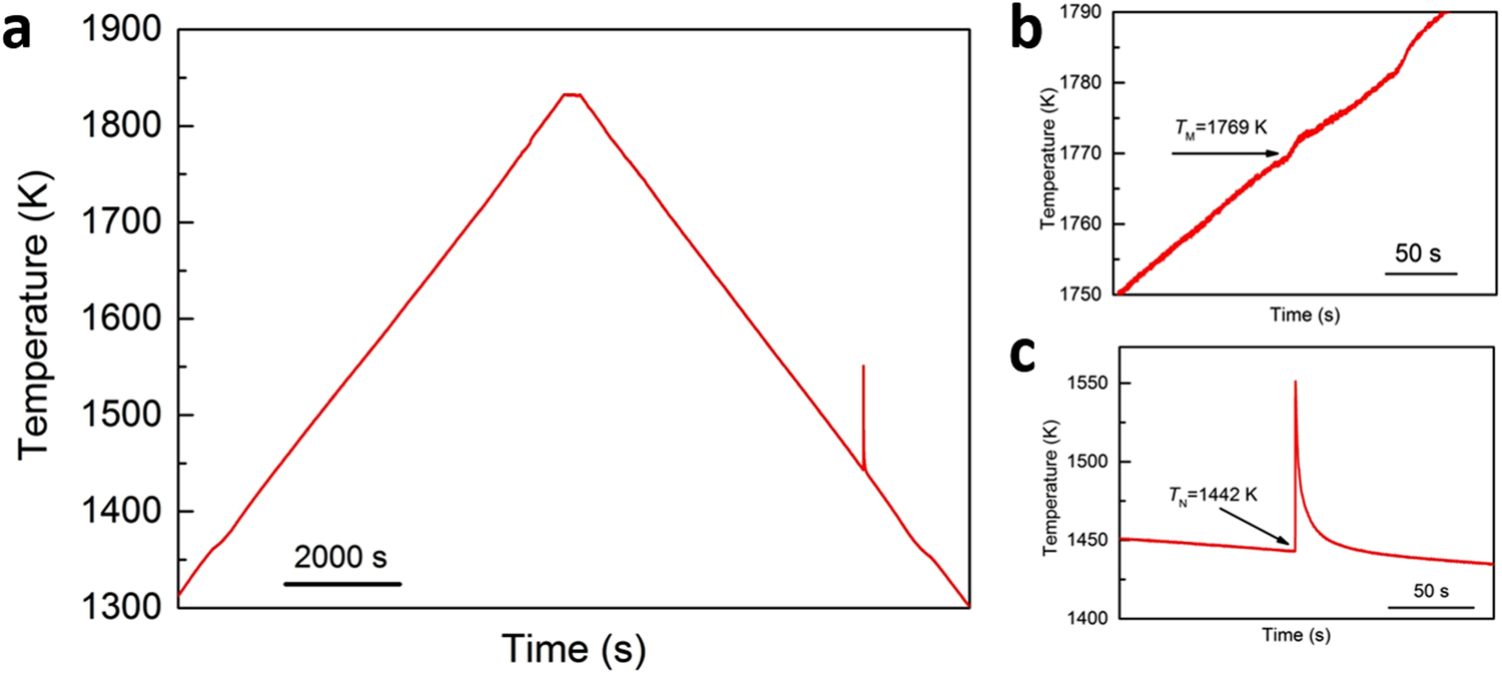
(**a**) Temperature profiles of Co during heating and cooling, and enlarged area showing the (**b**) melting and (**c**) solidification temperature.

Many methods have been adopted to receive high undercooling of Co, e.g. levitation (232 K^16^), drop tube (330 K^17^), glass fluxing (300 K^12^ and 336 K^13^). During the present measurement, the experiments are conducted at controlled constant heating and cooling rate. A typical heating curve is shown in Fig. 1a. The sample is heated at constant rate about 5 K/min to about 1833 K and holding for 10 min, then cooling down to 1273 K. The undercooling, Δ*T*, is determined by the difference between the melting point (discontinuous point during heating, *T*_M_, 1769 K in Fig. 1b and nucleation point (rapid rise of temperature showing the release of latent heat due to the strong undercooling in the cooling process, *T*_N_, 1442 K in Fig. 1c). The stable undercooling (keep almost constant value consecutively for more than 10 times) of Co can reach to about 329 ± 10 K with and without magnetic field, indicating a very good undercoolability compared with other methods.

According to the alloy database, the *T*_C_ of Co is about 1394 K, indicating the liquid will be in ferromagnetic state (in case the ferromagnetic transition on the solid and liquid is the same) if the temperature of undercooled melt bellows 1394 K. During our previous measurement, strong magnetized liquid is observed when close to *T*_C_^13^. Then if it is possible to have ΔT > 373 K (*T*_N_ = 1394 K), the nature of the liquid will be totally different from above. Since we are close but still above *T*_C_, this case will not be discussed in the present study.

### The magnetization of liquid Co within a wide temperature range in different magnetic fields

**Figure 2:**
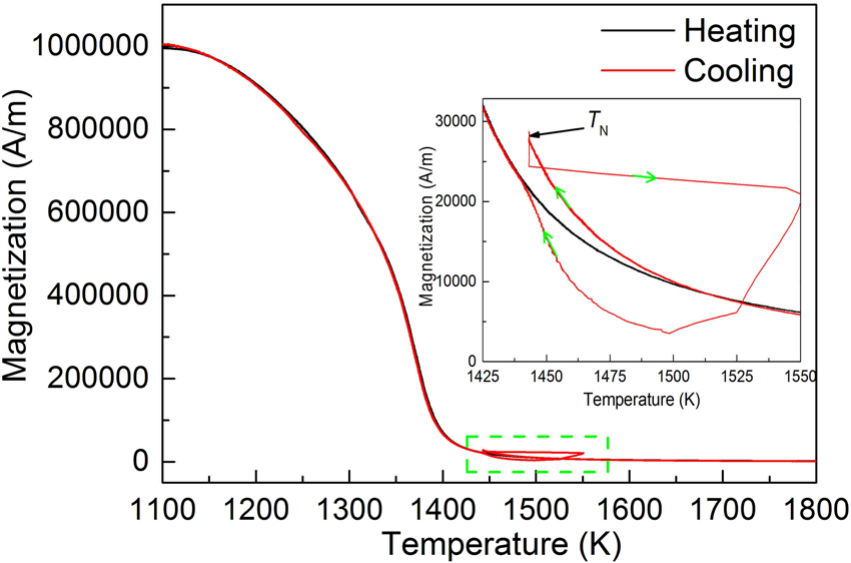
The magnetization of Co measured during heating and cooling process in 0.52 T magnetic field and field gradient of 7.8 T/m. Inset is the enlarged area showing the magnetization variations around the nucleation point.

A typical magnetization-temperature curve of pure Co is shown in Fig. 2. During heating process, the magnetization decreases with increasing temperature and a catastrophic decrease happens when approaching *T*_C_. Because of the existence of remaining short range order above *T*_C_, the magnetization curve always reveals a remaining tail in an external magnetic field, which progressively decreases to regular paramagnetism. When the temperature is above *T*_C_, the metal is in paramagnetic state, and the magnetization decreases continuously with the increasing temperature. During the cooling process, the magnetization increases with the decreasing temperature. Then the alloy melt comes into the undercooled state. The magnetization of the undercooled melt is almost overlapped compared with the magnetization curve during heating. When the temperature bellows 1520 K, the magnetization of the undercooled liquid starts to increase faster, as seen the inset figure in Fig. 2, indicating the magnetization of the liquid is higher than the solid at the same temperature. The difference increases with the increasing undercooling before the nucleation temperature at 1443 K. The magnetization of the undercooled liquid at 1443 K, *M*_liquid_ = 27600 A/m, which is about 30% larger than that of the magnetization of solid where *M*_solid_ = 21500 A/m. Due to the rapid temperature increase in the recalescence process, the balance can not accurately measure the data due to its slow stabilization time for receiving a correct data in the fast solidification process. However, we still can see the trend: rapidly decrease until the maximum temperature after nucleation, which is consistent with the temperature measured by pyrometer. After the rapid solidification progress is finished, the magnetization curve comes back to the value measured during heating process when the temperature slows down to 1443 K again.

Above *T*_C_, a ferromagnetic material becomes paramagnetic and obeys the Curie-Weiss law as:

1 $$\chi =\frac{C}{T-{\theta }_{P}}$$

where χ, *C* and *θ*_p_ are magnetic susceptibility, Curie constant and the asymptotic or paramagnetic Curie temperature, respectively.

Using *M* = *χH*, equation (1) can be changed to:

2 $$M=\frac{C}{T-{\theta }_{P}}\cdot H$$

**Figure 3:**
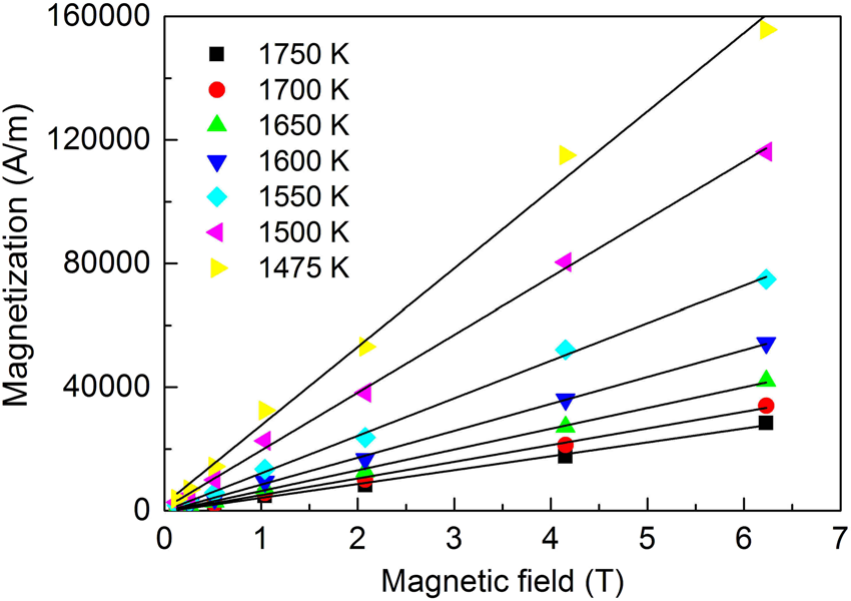
The magnetization as a function of magnetic field intensity of liquid Co measured at different undercooling temperature.

Thus, at constant temperature, the magnetization is a linear function of magnetic field intensity when Curie-Weiss law is applicable. The magnetizations at different temperature of the liquid Co (undercooled state) under different magnetic field intensities are shown in Fig. 3. It can be seen that the magnetization can be treated has a linear relation at the whole undercooling range of the melt (the linear treatment will be not true at very high magnetic field or high undercooling very close to the *T*_C_ of liquid).

By inserting the determined parameters into equation (2), the magnetization of Co above Curie temperature are calculated for solid during heating and liquid during cooling, respectively.

Solid Co during heating

3 $$M=\frac{2.072}{T-1408}\cdot H$$

Liquid Co during cooling

4 $$M=\frac{1.81}{T-1421}\cdot H$$

**Figure 4:**
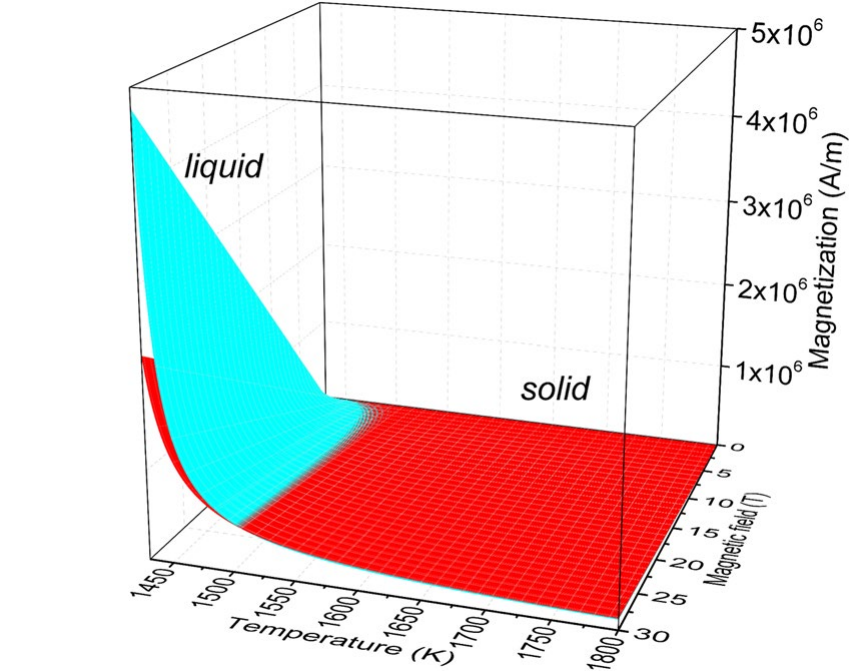
The calculated magnetization as a function of magnetic field intensity and undercooling temperature for solid Co during heating and liquid Co during cooling.

Then the magnetization of solid and liquid Co in a wide temperature range and different field intensities can be calculated which are shown in Fig. 4. The magnetization of the solid and liquid is very close at high temperature (low undercooling) and low magnetic field, a critical line is observed showing that the liquid metal becomes much more magnetic than solid at the same undercooling and field intensity. The magnetization difference above the critical line is larger when the undercooling and field intensity is higher. The intersection line will turn to high temperature (low undercooling side) with the increasing field intensity, indicating the magnetic field affects the magnetic state of liquid and solid Co.

### The critical size and magnetization for instability

The high magnetization of the liquid then allows surface instabilities that frozen in the resulting solid. This instability, as studied in ferrofluids, is governed by the competition between demagnetizing energy that promotes the formation of peaks, and the surface tension and gravity that tend to smooth and flatten the surface. The interspacing is also strongly dependent on gravity, as will be discussed later. Following Cowley and Rosensweig^18^, the onset of the instability is related to two critical parameters: the critical spacing between peaks λ_C_ and the critical magnetization *M*_C_ with ref. 7:

5 $${\lambda }_{C}=2\pi {(\frac{\sigma }{g{\rm{\Delta }}{\rho }_{m}})}^{\frac{1}{2}}$$

6 $${M}_{C}^{2}=\frac{2}{{\mu }_{0}}(1+\frac{1}{{r}_{0}}){(g{\rm{\Delta }}{\rho }_{m}\sigma )}^{\frac{1}{2}}$$

where σ denotes the interfacial tension, *g* the gravitational constant, Δ*ρ*_m_ the difference in specific mass of the liquids across the interface, *μ*_0_ the permeability of vacuum, and *r*_0_ the dimensionless permeability ratio that can be defined as:

7 $${r}_{0}={(\frac{{\mu }_{c}{\mu }_{t}}{{\mu }_{0}^{2}})}^{\frac{1}{2}}$$

where *r*_0_ depends on two permeabilities at the operation point, the chord permeability *μ*_c_, and the tangent permeability *μ*_t_, and *r*_0_ can be estimated to be 1.

In our experiments, the vertical force per unit mass of the sample is not negligible as compared to gravity. In the above equations, the force per unit volume *g*Δ*ρ*_m_ for the Co sample should then be replaced by (*g*Δ*ρ*_m_ + *MdB*/*dz*). The new set of equations, related to λ_C_ and *M*_C_, then becomes:

8 $${\lambda }_{C}=2\pi {(\frac{\sigma }{g{\rm{\Delta }}{\rho }_{m}+M\frac{dB}{dz}})}^{\frac{1}{2}}$$

9 $${M}_{C}^{2}=\frac{2}{{\mu }_{0}}(1+\frac{1}{{r}_{0}}){((g{\rm{\Delta }}{\rho }_{m}+M\frac{dB}{dz})\sigma )}^{\frac{1}{2}}$$

In order to calculate the critical diameter and magnetization for instability, several parameters need to be known first. With the increasing undercooling, parameters, e.g. σ, ρ will be changed, thus it will be helpful to express them as a function of temperature.

The solid-liquid interfacial energy σ(T) depends on the temperature can be determined using negentropic model^19^, which can be expressed as:

10 $$\sigma (T)=\frac{\alpha {\rm{\Delta }}{S}_{f}T}{{N}_{A}^{1/3}{V}_{m}^{2/3}}$$

where α = 0.86 is the Turnbull constant for BCC crystal structure, ΔS_f_ is entropy of fusion (9.08 J·mol^−1^·K^−1^) and *V*_m_ is the molar volume of Co (7.23 × 10^−6^ m^3^/mol).

The density of solid Co during heating and liquid Co during cooling, can be calculated using the following expressions^20^:

11 $${\rho }_{solid}=\frac{{\rho }_{0}}{(1+3\alpha (T-{T}_{0}))}$$

where ρ_0_ is the density of the solid metal at *T*_0_ and α is the thermal expansion coefficient (14 × 10^−6^ K^−1^) of the material.

12 $${\rho }_{liquid}={\rho }_{0}+(T-{T}_{m})\frac{d\rho }{dT}$$

where ρ_0_ is the density of the liquid metal (7.76 g/cm^3^) at its melting point, and dρ/d*T* is −0.988 mg/(cm^3^K) for Co.

**Figure 5:**
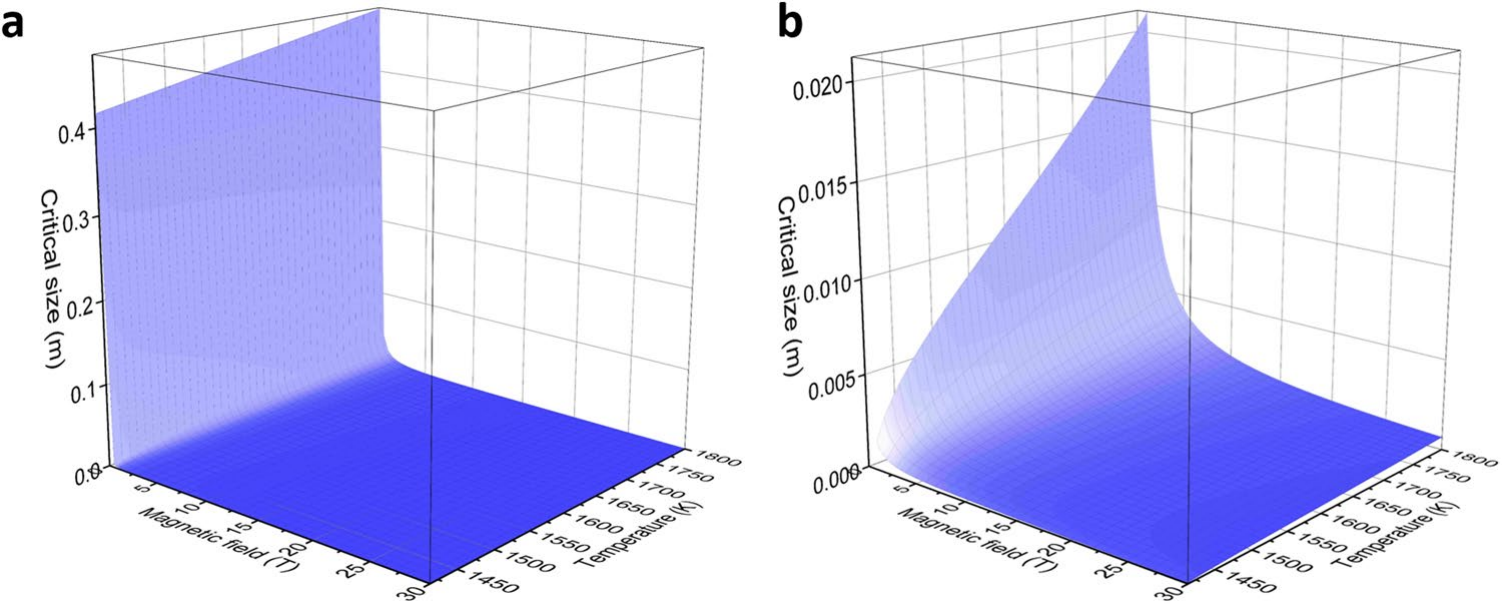
The critical size for instability formation of liquid Co as a function of temperature and magnetic field intensity (**a**) and (**b**) enlarged area when the field intensity is above 1 T.

**Figure 6:**
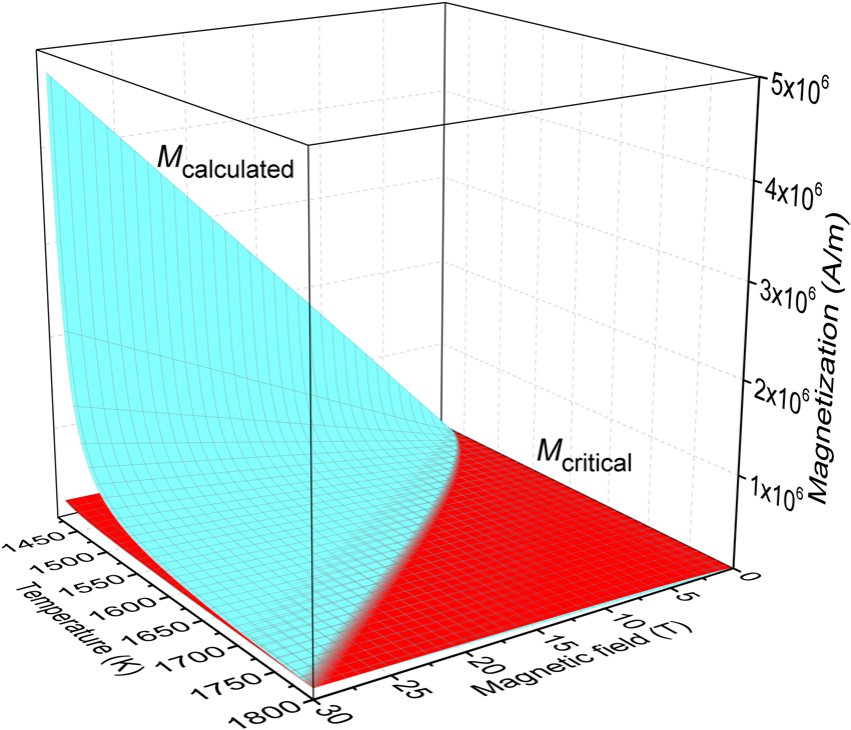
Experimental magnetizations and critical magnetization for the existence of normal-field instability in liquid Co alloy.

Then the critical sample size (*λ*_C_) and critical magnetization (*M*_C_) can be obtained by the above equations, as shown in Fig. 5 and Fig. 6. When the field intensity is very low (<1 T), seen from Fig. 5a, the critical size for instability formation is above 2 cm and even can be up to 50 cm, which is impossible to have instability pattern due to the difficulty to obtain high undercooling of the sample. The critical size of the sample is relatively acceptable in case when the field intensity is relatively large, e.g. >1 T, and the critical size can be constrained to a few mm which allows the supercooling of the metal, as shown in Fig. 5b. The critical size is just 1.5 mm in 1 T magnetic field when the liquid temperature is 1430 K (ΔT = 339 K) while it increases to 21 mm when the temperature is 1800 K. At the same temperature of 1430 K, the critical size changes from 1.5 mm to 0.05 mm when the field intensity is increased from 1 T to 30 T. Both increasing magnetic field intensity and undercooling can reduce the critical size. According to Fig. 5, in case when the field intensity is larger than 1 T and the melt is in undercooled state, the critical size is not a decisive parameter to obtain the instability pattern.

The decisive parameter for the instability pattern formation is the critical magnetization (*M*_C_), since the critical size means nothing when the magnetization is below the critical value. *M*_C_ for the existence of Rosensweig instability is also strongly related with the field intensity and undercooling. When the undercooling or field intensity is not large enough, the critical magnetization will increase slowly. However, dramatic increase will happen in case the undercooling and field intensity approach to the maximum of the present calculation. At low undercooling or low magnetic field, the magnetization of the liquid Co will be very small (shown in Fig. 4). In this case it is impossible to observe the instability pattern.

### The critical condition for instability in liquid Co

When we compare the results between the calculated experimental results and critical magnetization, the required condition for the instability pattern can be easily obtained, as shown in Fig. 6. An intersection curved line is clearly observed, which can be treated as the critical condition required for Rosensweig instability pattern formation. The two boundaries of the intersecting line are determined first. At very low field, the starting point lines in high undercooling range. It means nothing when there is no magnetic field, thus we choose a low field, e.g. 1 T, as the starting point where the nucleation temperature is about 1461 K (ΔT = 308 K). The other point is in 30 T magnetic field, where the nucleation temperature is about 1768 K (ΔT = 1 K). Then we consider the effect of the critical size, since large undercooling cannot be reached when the sample size is too big. Under high magnetic field, the undercooling required is very small, and the critical size, at 1768 K is about 0.6 mm (field intensity: 30 T), which is quite easy to obtain undercooling larger than 1 K. The critical size at 1461 K (field intensity: 1 T) is about 5 mm, which is still allowed to obtain high undercooling as 315 K. Thus considering the real experimental difficulty, Fig. 6 can be used to determine the critical conditions for the instability pattern formation in liquid Co.

## Discussions

**Figure 7:**
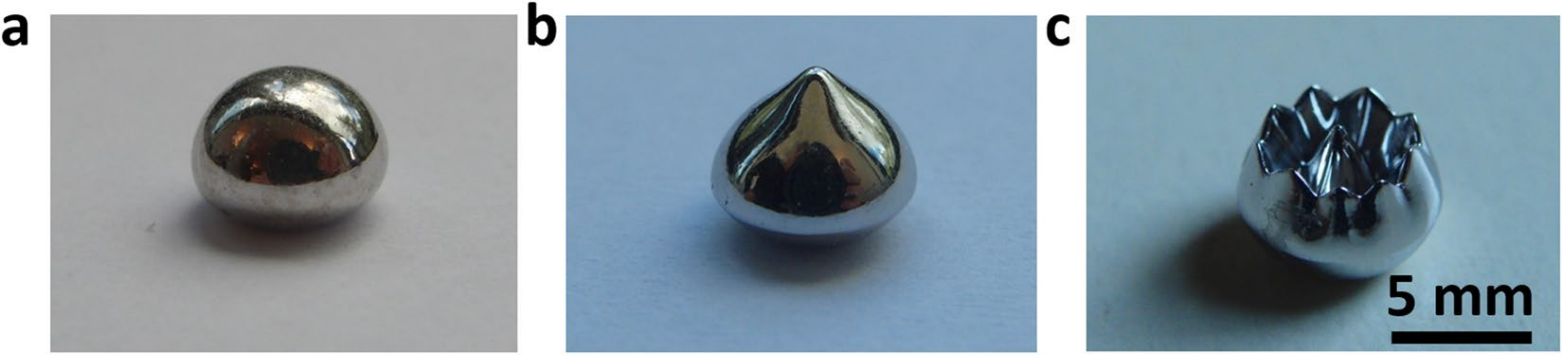
Instability pattern morphology formed by undercooled liquid Co under different magnetic fields, (**a**) wihtout magnetic field, (**b**) 1 T magnetic field and a field gradient 15 T/m and (**c**) 4 T magnetic field and a field gradient 60 T/m.

The critical length *λ*_C_ is the distance between the two peaks of the sample. To distincly evidence this kind of pattern, the sample size needs to be at least two times the length of the critical size. Considering for the diameter of crucible is about 6-7 mm for the present experiment, then the critical length *λ*_C_ should be smaller than 3 mm. The stable maximum nucleation temperature that can be controlled is about 1438 K (ΔT = 329 K). Take Fig. 5 into condieration, *λ*_C_ is about 3 and 2 mm for the samples processed at 1 and 2 T magnetic field, respectivelly. Figure 7 is the Co samples solidified at stable undercooling about 329 ± 10 K under different magnetic field instensities. Wihtout magnetic field, the sample is in spherical shape (Fig. 7a), and a main peak in the top part of the sample is observed when the sample solidified at 1 T magnetic filed (Fig. 7b). When the field increased to 4 T, shown in Fig. 7c, lotus like patterns with peaks and valleys are fomed, which is similar to the well known normal field instability observed at room temperature in ferrofluids^4, 5^.

At constant undercooling, with the same sample size, the critical magnetization will increase with the field intensity while the magnetization of the liquid increased much faster, especially when the temperature is below the critical undercooling.

During the calculation of the experimental magnetization of liquid Co, the undercooling is just restrained to about 339 K which is very close to the maximum undercooling. The maximum field intensity considered is about 30 T which is much higher than the experimental we have done (6 T), but it is possible to have in the lab. In case when the undercooling is large (very close or even below *T*_C_) or the field intensity is above certain value, the liquid Co will be saturated, which means the calculation in Fig. 4 will be wrong. Thus we will discuss about the saturation of liquid Co both considering the undercooling and field intensity.

**Figure 8:**
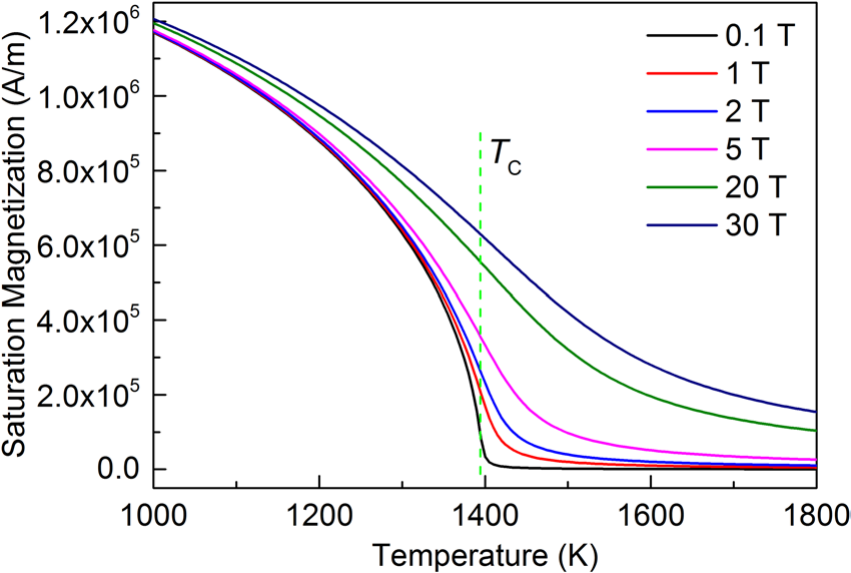
Saturation magnetization of Co calculated by mean field theory.

The saturation magnetization of Co (in solid state) is calculated using mean field theory. The magnetic field used for calculation ranges from low field from 0.1 T to high field as 30 T. The magnetization of liquid Co is higher than solid when the temperature is below certain value. That is to say, the saturation magnetization should be higher than the value calculated using the data of solid Co, as shown in Fig. 8. A linear relation can be found for the magnetization above *T*_C_ when the field intensity is not very high. At high field, the magnetization will not increase linearly with the field intensity, but we can still observe the increasing trend. When we take a comparison between the calculated magnetization at the undercooling of 339 K (shown in Fig. 6), it can draw the conclusion that the value in Fig. 8 is higher than the critical magnetization, indicating the liquid will be much higher. Thus even the linear assumption for the calculation of liquid Co is not correctly at high field or undercooling, but it still acceptable to determine the right value for the critical magnetization since the alloy is still not saturated by the magnetic field, and the critical magnetization required is much smaller than the real magnetization of the liquid.

The Rosensweig instability widely exists in ferrofluids. According to our calculation, it can be seen that, for the existence of normal field instability in pure liquid Co, two parameters, field gradient (dB/dz) and undercooling (ΔT) are required. Without undercooling, the liquid Co melt will be in paramagnetic state and the magnetization of the melt will be very small even high magnetic field is applied. Thus increasing undercooling will make the liquid much more magnetic at the same magnetic field, since ordering structure will form which prevails high magnetization.

## Conclusions

In summary, Rosensweig instability can be evidenced in pure liquid Co. The formation condition of the instability is discussed based on the calculation of the critical magnetization and wave length, in comparison with the calculated experimental value. The critical condition for the instability is determined by considering the undercooling and field intensity, and large undercooling and high magnetic field are in favor of the formation of normal field instability.

## Methods

### Sample preparation and undercooling procedure

Samples of pure Co were cut in a cuboid shape with mass value about 1 g directlly from cobalt plates (99.99 wt%). The sample is encapsulated in B_2_O_3_ glass and heated in an open air high purity quartz tube. The glass fluxing and cyclic heating methods is conducted to obtain supercooling of the Co liquid. A special furnace was designed to fit inside the 50 mm room temperature bore of a cryogen free superconducting magnet, with a silicon carbide heating element that can resist the electromagnetic forces in the magnetic field and a water cooled jacket to protect the magnet from heat. The sample temperature is monitored by a bichromatic pyrometer. In order to make good measurements with high accuracy and reproduceble, all the measurements are carried at the same thermal program especially at same heating and cooling rate about 5 K/min, in order to reduce the temperature gradient and the error for magnetization measurement. The real measurement is taken after the melt is in a thermal stable state when the maximum undercooling keeps stable at certain value.

### Measurement for the magnetic force

The sample is placed above the maximum field of the magnet, at a position where the radial magnetic forces will center it on the axis on the magnet. The vertical magnetic field gradient exerts a downward vertical force that is monitored by a electronic balance placed above the magnet and below which the quartz crucible is suspended. The sample magnetization is then continuously monitored from this force measurement during the melting, overheating and solidification process. Detailed description of the apparatus is shown in ref. 21.
